# Density Dependence Triggers Runaway Selection of Reduced Senescence

**DOI:** 10.1371/journal.pcbi.0030256

**Published:** 2007-12-28

**Authors:** Robert M Seymour, C. Patrick Doncaster

**Affiliations:** 1 Department of Mathematics, University College London, London, United Kingdom; 2 Centre for Mathematics and Physics in the Life Sciences and Experimental Biology (CoMPLEX), University College London, London, United Kingdom; 3 School of Biological Sciences, University of Southampton, Southampton, United Kingdom; University of Sussex, United Kingdom

## Abstract

In the presence of exogenous mortality risks, future reproduction by an individual is worth less than present reproduction to its fitness. Senescent aging thus results inevitably from transferring net fertility into younger ages. Some long-lived organisms appear to defy theory, however, presenting negligible senescence (e.g., hydra) and extended lifespans (e.g., Bristlecone Pine). Here, we investigate the possibility that the onset of vitality loss can be delayed indefinitely, even accepting the abundant evidence that reproduction is intrinsically costly to survival. For an environment with constant hazard, we establish that natural selection itself contributes to increasing density-dependent recruitment losses. We then develop a generalized model of accelerating vitality loss for analyzing fitness optima as a tradeoff between compression and spread in the age profile of net fertility. Across a realistic spectrum of senescent age profiles, density regulation of recruitment can trigger runaway selection for ever-reducing senescence. This novel prediction applies without requirement for special life-history characteristics such as indeterminate somatic growth or increasing fecundity with age. The evolution of nonsenescence from senescence is robust to the presence of exogenous adult mortality, which tends instead to increase the age-independent component of vitality loss. We simulate examples of runaway selection leading to negligible senescence and even intrinsic immortality.

## Author Summary

Senescent aging is an irreversible deterioration in physiological condition with age, which many organisms express even when removed from harmful environmental influences. The inevitability of senescence for repeatedly reproducing organisms has well-developed theoretical foundations. Since reproduction carries physiological costs, natural selection in a hazardous environment favors reaping early benefits, and delaying the cost in physiological decline until later in life when there is a greater chance of being dead from exogenous factors. But some organisms show negligible senescence, and a few, such as *Hydra* and the Bristlecone Pine, appear to have indefinite lifespans. We ask how such species could have evolved from ancestors with senescent life histories. In large populations, juveniles attempting recruitment into the adult population can be “crowded out” by already established adults. We show how this phenomenon can trigger a process of runaway selection on ever-reducing senescence, which can even result in the evolution of intrinsic immortality. Contrary to previous hypotheses, we find the rate of senescence to be insensitive to environmental hazard, which instead influences background, age-independent rates of physiological decline.

## Introduction

Senescence, usually treated as synonymous with “aging,” refers to a deterioration in physiological condition with age, manifest as an increase in mortality and a decline in fertility. Since this phenomenon is detrimental to reproductive success, natural selection might be expected to cause its postponement or elimination from the life history of organisms. Its apparent ubiquity in the natural world, therefore, has been treated as a challenge for evolutionary theory [1]. There is a general acceptance that this challenge has, in principle, been met, and modern understanding of the widespread occurrence of senescence in nature has been hailed as one of the great triumphs of evolutionary thinking [2].

Discussions of the evolution of senescence broadly follow one of two paradigms, based either in classical population genetics or in physiological ecology [3]. The first emphasizes the accumulation of late-acting deleterious mutations on hypothesized genes with age-specific expression [1,4]. More generally, this paradigm hypothesizes an antagonistic pleiotropy of age-specific genes in which mutations confer a fitness benefit early in life at the cost of some deleterious effect later [4–6]. The key insight of this perspective is that the force of selection on the additive component of genetic variance necessarily declines with age, so that an early-age cost is more strongly selected against than an equivalent late-age cost, and, a fortiori, an early-age benefit more than compensates for a late-age cost [4]. Hamilton concludes: “...for organisms that reproduce repeatedly, senescence is to be expected as an inevitable consequence of the working of natural selection” [4].

This population genetic analysis has been challenged recently both theoretically and empirically. First, a declining force of selection is only guaranteed for mutations with additive effects, and it has been suggested that mutations with proportional effects—for which the force of selection need not decline with age—may be more relevant [7]. Second, Hamilton himself concedes: “To what extent and in exactly what way life schedules will be moulded by natural selection depends on what sort of genetical variation is available” [4]. Thus, the more such genes there are, the more evolutionary pressure there will be toward compressed life histories. However, despite much empirical work over several decades, evidence for the availability of genes with the necessary age-specific effects appears to be thin (reviewed in [8]).

In contrast to the classical population genetic approach, the “disposable soma” theory [3,9–11] is based firmly within physiological ecology. Thus, it is claimed that birth and death schedules are the result of the action of integrated physiological processes concerned with the optimal partitioning of available resources between reproduction and somatic maintenance or growth. In particular, there is an inherent “cost of reproduction” in which an early-age reproductive benefit incurs a late-age cost in decreased survival, possibly in the form of latent damage that is only unmasked later in life [8]. Relevant genetic mutations must have effects that are manifest at this physiological level. This is potentially a much more constraining paradigm, though apparently more strongly supported by current evidence [8,12]. Nevertheless, given this tradeoff between early fecundity and longevity, it has again generally been concluded that senescence is inevitable [3]. In particular, immortality has long been considered theoretically impossible because of the inevitability of senescent aging [13–16].

Yet this understanding of the evolution of senescence fails to account for organisms showing negligible or even negative senescence [16,17]. These are species such as the freshwater Hydra vulgaris [18] with period survival that remains constant or increases with adult age. They include some organisms with apparently indefinite lifespans such as the Great Basin Bristlecone Pine Pinus longaeva [19,20], which continues producing viable cones at well over 4,000 y old, the Quaking Aspen Populus tremuloides [21], and the Creosote Bush Larrea tridentata [22], both of which have clonal clusters at least 10,000 y old.

These and other examples [16] have recently led to a reversal of the traditional perspective in which the problem was to explain the evolution of senescence from nonsenescence. On the contrary, given the ubiquity of senescence in nature, and the abundance of explanations for its presence, it seems very unlikely that the majority of today's organisms are descended from nonsenescent ancestors (even bacteria exhibit senescence [23]). Rather, an important issue now is to provide an evolutionary account for those organisms that appear to exhibit little or no senescence, but which almost certainly have evolved from ancestors that did exhibit senescence.

Rising to this challenge, a theoretical analysis of the costs of senescent aging [24] has shown that, although senescence is often favored by a high and sustained early vitality (a measure of intrinsic net fertility), nonsenescing strategies are locally optimal if vitality loss in the presence of senescence would otherwise be sufficiently fast. Similarly, an optimization model [17] has shown how negative senescence can evolve for species that grow in body size throughout their lives, if this growth carries proportionate benefits in increasing reproductive output and decreasing mortality. Both these analyses are concerned with the optimal tradeoff between fecundity and mortality, and so lie within the disposable soma paradigm.

These analyses have not modeled density dependence, except implicitly as a limiting case in which population growth is set to zero. In this paper, we construct explicit models, within the disposable soma paradigm, for a very general class of organisms including those without indefinite somatic growth. These reveal density dependence in recruitment as a sufficient driver for the evolution of nonsenescent life histories from senescent ancestors. Density-limited recruitment sets up a balance of opposing selective forces that underpins the direction of evolution toward either compressed (shorter and faster) or spread (longer and slower) reproductive life. Thus, on the one hand, future reproduction is worth less than present reproduction to an individual's fitness, given a future extrinsic mortality risk [1]. On the other hand, future reproduction by an individual's mature offspring may be worth more to its inclusive fitness than its own present reproduction, if otherwise viable offspring face an extrinsic mortality risk before recruitment.

The crucial advance that we make is prefigured by Abrams [25], who showed that faster senescence is favored by positive or zero density-independent growth, and also by density-dependent adult mortality, whereas slower senescence requires density-dependent fecundity. Our advance on his analysis is to show how the slower senescence can take the form of a runaway selection to negligible senescence, and even intrinsic immortality. Indeed, density-dependent recruitment reflects the widely prevailing ecological condition of bottom-up regulation in crowded habitats. We show that it is unwise either to ignore it, or to represent it only implicitly as zero population growth, because of its ubiquity in nature and its significant consequences for the evolution of senescence.

Here, we perform an optimization analysis of vitality evolution as a fitness tradeoff between compression into earlier life and spread into later life in the context of density-dependent recruitment, which accords with the abundant evidence that reproduction is intrinsically costly to survival [8,13,15,26–28]. For populations at recruitment-regulated equilibrium, we demonstrate generic conditions under which natural selection itself increases the extrinsic recruitment losses, by successive genomic invasions increasing the level of crowding within the population. Stronger density dependence means fewer recruitment opportunities into the adult population and, therefore, a natural selection that is weighted toward maximizing generation length over early-age vitality. This positive feedback leads to the novel result that density regulation can trigger selection for ever-reducing senescence. We develop a model that shows the potential for runaway selection of reduced senescence to arise across a wide range of age-specific vitality profiles, including accelerating loss from an early or late onset, and constant aging (concordant with Deevey types I and II survivorship). We find that natural selection can favor evolution of nonsenescence, and even immortality, from senescence in the presence of exogenous mortality, without a requirement for special life-history characteristics such as increasing intrinsic fecundity with age (cf. [17]). Simulations of this process are given for various scenarios, including stochastic environments.

The first four Results sections develop our analytical framework. Section 1 outlines the assumptions we make about the action of density-dependent recruitment. Section 2 specifies precisely the relation between the concepts that we use of vitality and senescent and nonsenescent aging. Our approach is to define these concepts in terms of “instantaneous rates” (affecting a combination of mortality and fecundity; cf. [29]), rather than on the rate of change of age-specific reproductive value (e.g., [30]). The section Model: Invasion of Mutations outlines our assumptions concerning the effects of mutations on the key life-history parameter controlling the rate of senescence, and states our main result concerning the possibility of evolution from a highly compressed life history to a highly spread life history. The section Example describes a specific example of evolution from positive senescence to non- (or even negative) senescence. Finally, the section Simulations outlines stochastic simulations of the model. Supporting material is provided in the Methods section and in Text S1. We conclude with a discussion of the model predictions for life-history conditions and biotic environments that favor negligible senescence.

## Results

### Model: Density Dependence and the Euler-Lotka Equation

Consider a population of organisms in which juveniles reach reproductive maturity at age *a* = *a_m_*. For ages *a* ≥ *a_m_*, write *t* = *a* − *a_m_* for the relative age of an adult. Thus, individuals of age *t* = 0 are newly recruited adults. The total population density of adults is *N*. We assume that density-dependent effects have consequences for the juvenile phase as follows.

1) The probability of survival of a juvenile from birth to maturity is an adult-density–dependent function of the form *σ*_0_(*N*) = *f* · *ℓ*(*N*).
2) *f* is the juvenile survival probability in density-independent conditions.
3) *ℓ*(*N*) is a nonincreasing function of *N* satisfying *ℓ*(0) = 1.
4) The birth rate of adults of relative age *t* ≥ 0 is an adult-density–dependent function of the form 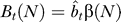 .
5) 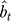 is the intrinsic, density-independent component of age-specific birth rate.
6) *β*(*N*) is an age-independent, nonincreasing function of *N* satisfying *β*(0) = 1.
7) *F*(*N*) = *ℓ*(*N*)*β*(*N*) is a decreasing function of *N* satisfying *F*(0) = 1 and *F*(*N*) → 0 as *N* → *K* (a fixed “carrying capacity,” which may be infinite).
8) The instantaneous mortality rate *d_t_* of adults of age *t* ≥ 0 is density-independent. This includes both intrinsic and extrinsic mortality factors.

Density dependence is assumed to act only through adult density on recruitment into the adult population, with the density of juveniles having no impact. For example, juveniles may be plant seeds, or small and mobile (e.g., planktonic), whereas adults are sessile and confined to specialized habitats. Density dependence then acts through one or both of two routes. Assumptions 1)–3) describe its possible action on juvenile survival. If there is no density dependence, juveniles survive from birth to maturity with maximum positive probability *f*. If density dependence acts, however, juvenile survival is reduced with increasing adult population via the decreasing function *ℓ*(*N*). This occurs in recruitment-limited populations, for example, if adults occupy a high proportion of potential recruitment sites. Assumptions 4)–6) describe the possible action of density dependence on adult fecundity. Thus, if there is no such action, age-dependent adult birth rates 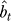 are given intrinsically. If there is a density-dependent effect, it is assumed to act uniformly on all adult age classes through the age-independent decreasing function *β*(*N*). For example, competition between increasingly many adults for limited nutritional resources may lead to a decrease in metabolic capacity available for reproduction beyond what is required for somatic maintenance. It is unlikely in reality that such effects would be age independent, but we assume this to be approximately the case for the sake of technical simplicity. Assumption 7) says that at least one, and possibly both, of these two density-dependent effects operates, and that their combined effect has the potential to reduce adult recruitment to zero at sufficiently high adult population densities (at which point the population reaches its carrying capacity *K*). This clearly has a self-limiting effect on adult population size. Finally, for technical simplicity, we assume that there is no density-dependent effect on adult mortality (assumption 8)).

These assumptions restrict the action of density dependence to a net effect on the recruitment of juveniles to the adult population. We focus on density-dependent recruitment because it is a prevailing ecological condition, and because others have previously shown it to favor slower senescence [25] and longer lifespan [31]. We suspect that natural selection may respond to the wastage of offspring that have low recruitment probability by spreading out the production over a longer lifespan. Since fitness-raising mutations raise adult carrying capacity, they always intensify this wastage in density-dependent juvenile losses, leading us to hypothesize the possibility of a runaway process.

The probability of survival of adults from maturity to age *t* ≥ 0 is:

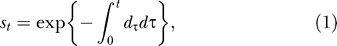
which is density-independent.

Suppose the population is at (age-structured) equilibrium, with equilibrium adult population density N^*^. Then it is easily shown that the equilibrium Euler-Lotka equation holds:

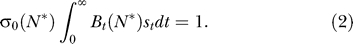



Write *b_t_* = 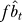 . Then, *b_t_* is the density-independent rate of recruitment of new adults (age *t* = 0) whose parent was of age *t* when they were born. In view of assumptions 1)–7), we may therefore write Equation 2 in the form:

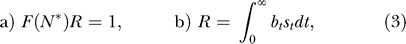
where *F*(*N*) = *ℓ*(*N*)*β*(*N*) is a decreasing function of *N* satisfying *F*(0) = 1, and *F*(*N*) → 0 as *N* → *K. R* is the expected lifetime reproductive success (ELRS) of an adult in the absence of density effects (which act only through juveniles). For a positive equilibrium population density N^*^, we have *F*(N^*^) < 1, and hence *R* > 1 from Equation 3a. The larger N^*^ is, the larger *R* must be to sustain Equation 3a. That is, equilibrium population density is an increasing function of *R*.

### Model: Senescent and Nonsenescent Aging as Components of Vitality Loss

Intrinsic characteristics determine the organism's ability to effect somatic and genetic repair, and generally to maintain its condition in the face of challenges inherent in its genetic makeup and environment. Extrinsic characteristics, which generally influence mortality, are determined by residual features of the environment, such as fluctuations in resource availability, a sudden spike in predator numbers, and various possible accidents such as floods or drought, etc. We regard an organism as susceptible to cumulative damage throughout its life, such as damage to DNA from free radicals [32], but also as having some capacity to repair this damage. In particular, we regard growth and development as a process in which an organism not only can repair the damage it sustains, but also has excess “capital” to invest in the development of additional soma. The rate of “senescence” can be thought of as the net rate at which damage accumulates. Thus, senescence sets in when the organism can no longer repair all the damage it sustains, and physiological deterioration results: that is, rate of damage accumulation is greater than rate of repair. Negative senescence is the opposite: rate of damage accumulation is less than rate of repair. In this case, somatic growth leads either to increased reproductive output, or decreased mortality, or both (cf. [17]). A classic life-history trajectory begins with a new adult organism exhibiting either no or negative senescence (increasing size), which later declines with age until accumulated damage outstrips the organism's capacity for repair, and (increasing) senescence sets in at older ages.

To formalize these points, we first decompose mortality into intrinsic and extrinsic components:

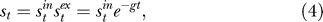
where we have assumed (for simplicity) that the extrinsic mortality rate *g* is constant. For fecundity, we write

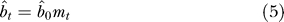



where 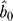 is the birth rate of newly recruited adults (age *t* = 0), and *m_t_* is the intrinsic relative fecundity of adults of age *t*. Clearly, *m*_0_ = 1, and more generally, *m_t_* determines the proportion of new-adult fecundity that is retained by adults of age *t*. (This may be greater than one if fecundity of adults increases with age—a negative senescence effect.) Multiplying both sides by the density-independent juvenile survival rate *f* gives a decomposition of adult recruitment rates: *b_t_* = *b*_0_ *m_t_*.

Following [24], we combine these characteristics and their interactions in the notion the intrinsic relative vitality of adults of age *t*, defined by:

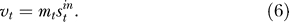



Clearly, *v*_0_ = 1, and more generally relative vitality measures how well an organism of age *t* > 0 can be expected to survive and reproduce relative to its performance as a new adult (age *t* = 0). The ELRS Equation 3b can now be written in the form:

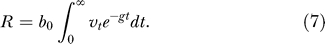



Now define two associated quantities. First, the age-specific rate of loss of vitality:

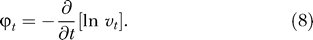
This allows us to write:

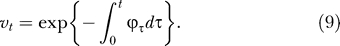
If *ϕ_t_* > 0, then relative vitality *v_t_* declines with age. This is to be expected due to the inherent cost of reproduction—any increase with age in relative fecundity is assumed to be more than offset by an intrinsic mortality cost. Senescence is the familiar accelerating loss of vitality that results from damage accumulation exceeding repair. For early-adult ages, we will also allow the possibility of decelerating vitality loss when repair exceeds damage accumulation, which is negative senescence.

The point of inflection between positive and negative senescence is nonsenescent aging when repair just matches or counterbalances damage accumulation. This condition of sustenance (sensu [29]) results in constant vitality loss with age. A Deevey Type II (linear) survivorship profile resulting from constant intrinsic mortality is an expression of nonsenescent aging. Even in the absence of senescence (*ϕ_t_* constant), the organism retains a finite intrinsic lifespan determined by aging (*ϕ_t_* > 0). Only if there is no such aging does the organism become intrinsically immortal. We recognize that evolutionary theory has generally treated “aging” and “senescence” as synonyms. Nonsenescence is clearly not synonymous with intrinsic immortality, however; hence, our treatment of nonsenescent aging (or “aging”) as the age-independent component of vitality loss that precludes immortality.

In terms of the age-specific vitality loss *ϕ_t_*, we define the senescence rate to be 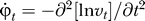 ; that is, by the acceleration (or deceleration) of the decline in relative vitality. Then we have:

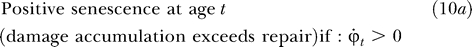


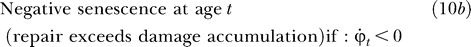


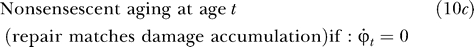



How senescence varies over an adult life history, and the evolutionary forces affecting this, will be investigated in subsequent sections.

### Model: Invasion of Mutations

In the simplest case, the rate of senescence is constant (age-independent), and can be specified in terms of a parameter *x*:

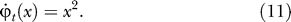
This gives a constant rate of positive senescence (see Equation 10a) when *x* > 0, but gives nonsenescent aging (see Equation 10c) when *x* = 0. More generally, the senescence rate 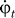 may be age-dependent, and then the senescence parameter *x* ^2^ can be defined as the asymptotic rate of senescence as *t* → ∞; i.e., the rate of (necessarily nonnegative) senescence at very old ages. These more-complex scenarios are considered in Appendix A of Text S1, which also treats the influence of negative senescence.

The age-specific rate of loss of vitality derived from Equation 11 is *ϕ_t_*(*x*) = *x* ^2^ *t* + *μ*_0_, where we interpret the constant component *μ*_0_ as the nonsenescent component of intrinsic mortality rate. Thus, from Equation 9, relative vitality declines with adult age according to the Gaussian:

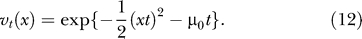



**Figure 1:**
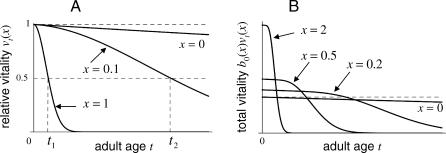
Graphs of Declining Vitality with Age (A) Decline in relative vitality with adult age given by Equation 12. For large *x* (*x* = 1), half the new-adult vitality is lost by an early age *t*_1_, whereas for small *x* (*x* = 0.1), it takes until age *t*_2_ to lose half the new-adult vitality. The curve *x* = 0 shows no senescence. (B) Decline in total vitality showing delayed-onset senescence, and also the tradeoff between senescence and new-adult fecundity, represented by the new-adult recruitment rate *b*_0_(*x*). The curve *x* = 0 shows no senescence. Here, the senescence rate is age dependent: 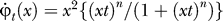 , with *n* = 5, and *b*_0_(*x*) = 1 + *x* (further detailed in Appendix A of Text S1). In both panels, the nonsenescent mortality rate is *μ*_0_ = 0.01.

With this form, a large *x* determines early onset of significant decline in relative vitality shortly after adulthood, whereas a small *x* delays significant vitality loss to later ages (see Figure 1A). Delayed-onset vitality loss—i.e., the maintenance of a high level of relative vitality for a significant proportion of life history—can also be represented by age-dependent forms of the senescence rate, as discussed in Appendix A of Text S1. An example is illustrated in Figure 1B.

The variable *x* determines the rate of senescence, so that an organism with a life history that maintains (iteroparous) reproductive activity over an extended lifespan has small *x*, whereas an organism with big-bang (semelparous) reproduction followed by an early death has large *x*. We assume that there is a fundamental tradeoff between early-adult reproduction and extended high relative vitality. Thus, we suppose that the new-adult birth rate 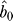 (and hence, the new-adult recruitment rate *b*_0_ = 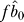 ) is an increasing function of *x*, so that high early-adult reproductive output (large *x*) is paid for by a rapid decline in relative vitality with age. Conversely, a low level of early-adult reproductive output (small *x*) may be sustained over a long intrinsic lifespan. From Equation 7, we can therefore write the ELRS as a function of the senescence parameter *x*:

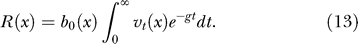



This tradeoff is illustrated in Figure 1B.

We assume that the evolution of this life-history tradeoff occurs through mutations in the senescence variable *x*. Given this assumption, the following invasion theorem holds (in accordance with [31,33,34]).

*Invasion Theorem:* a mutation that changes intrinsic adult life-history characteristics through variation in *x* will invade and go to fixation if and only if it results in an increase in the ELRS *R*(*x*).

This result depends on strong assumptions, in particular that the environment is constant and the intrinsic mortality component *μ*_0_ remains constant. In addition, it assumes that once a mutation has invaded a population, there will be sufficient time subsequently for it to go to fixation before some other, possibly compounding, mutation arises. These assumptions imply that our equilibrium populations are genetically homogeneous with respect to the relevant phenotypic expressions. This allows us to develop a simple analytical theory. However, all these assumptions will be relaxed in the computer simulations described in the section Simulations and in Appendix B of Text S1.

The equilibrium Euler-Lotka Equation 3a implies that an increase in *R* is equivalent to an increase in equilibrium density N^*^. This means that a mutant will invade a wild-type population if and only if the equilibrium density of a monomorphic population of mutants exceeds the equilibrium population density of the wild type.

We distinguish two opposing types of mutation, which we call a spreader mutation, which decreases *x*, and a compressor mutation, which increases *x*. The major effect of a spreader mutation is to increase the nominal transition age between “younger” (high-vitality) and “older” (low-vitality) adults (Figure 1). Thus, the intrinsic death rate decreases at all ages, promoting extended lifespan, and the birth rate decreases, at least initially, but may increase later in life, because the “young adult” birth rate profile is sustained for longer, albeit at a lower level. In effect, spreader mutations spread vitality into later life at a cost of a slower rate of reproduction. A compressor mutation has the opposite effect, increasing birth rates early in adulthood, but compensating by decreasing them later and increasing (particularly late-age) death rates. In effect, compressor mutations increase early-age vitality at a cost of an earlier onset and more precipitous senescent decline later in life in both fecundity and survival.

A mutation of small effect in the senescence variable *x* takes the form *x* → *x* + *δx*, where *δx* is a small change, which is negative for a spreader mutation (provided *x* > 0), and positive for a compressor mutation. Thus, from Equation 13, the mutation changes *R*(*x*) to *R*(*x* + *δx*) = *R*(*x*) + *R′*(*x*)*δx*. By the Invasion Theorem, the mutation will go to fixation provided *R*(*x* + *δx*) > *R*(*x*), i.e., provided *R′*(*x*)*δx* > 0. For a spreader mutation (*δx* < 0), this requires *R′*(*x*) < 0, and for a compressor mutation (*δx* > 0), it requires *R′*(*x*) > 0.

We shall show that it is possible, starting with a population that exhibits early-onset senescence (large *x*), for an indefinite sequence of spreader mutations to invade and go to fixation, resulting in an ever-increasing equilibrium population density N^*^ and an ever-increasing *R*.

As discussed above, we assume that the new-adult recruitment rate *b*_0_(*x*) is increasing in *x*. We also assume that the minimum new-adult recruitment rate, *ω*_0_ = *b*_0_(0), is positive, so that some reproductive output is achieved even in the nonsenescent state (*x* = 0). From Equation 12, relative vitality *v_t_*(*x*) is decreasing in *x* for each *t*, and therefore, 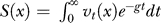 is also decreasing in *x*. Then from Equation 13, *R*(*x*) = *b*_0_(*x*)*S*(*x*) expresses the tradeoff between increasing early-adult reproduction and decreasing vitality with age. Clearly, *S*(*x*) “wins” this tradeoff—resulting in a decreasing *R*(*x*)—if *b*_0_(*x*) does not increase too fast with *x*. In effect, a slow increase in *b*_0_(*x*) expresses a strong inherent cost of early reproduction. This is the situation that favors invasion by spreader mutations. Conversely, *b*_0_(*x*) “wins” the tradeoff—resulting in an increasing *R*(*x*)—if *b*_0_(*x*) increases rapidly with *x*. This circumstance favors invasion by compressor mutations.

### Example

We seek new-adult recruitment functions *b*_0_(*x*) for which *R*(*x*) is decreasing in *x*. Consider the two-parameter family of near-linear functions:

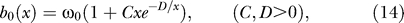



**Figure 2:**
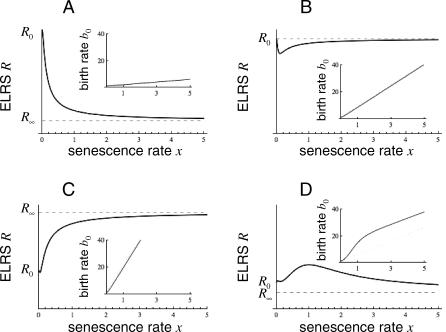
Graphs of *R*(*x*) as Functions of the Senescence Variable *x* for Various Initial Recruitment Functions *b*_0_(*x*) Graphs of *b*_0_(*x*) are shown in the inset panels. (A) As in Equation 14 with *C* = 1 and *D* = *μ*. In this case, *R*(*x*) is monotonically decreasing in *x*, and the evolutionary optimum is at the nonsenescent state *x =* 0. (B) As in Equation 14 with *C* = 8 and *D* = *μ*. In this case, *R*(*x*) has a minimum at a positive value of *x*. (C) As in Equation 14 with *C* = 20 and *D* = *μ*. In this case, *R*(*x*) is monotonically increasing, and the evolutionary optimum is the most extreme compressed life history, *x* = ∞. (D) A recruitment function not in the class of Equation 14: 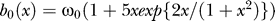 . In all cases, *ω*_0_ = 1, *μ* = *μ*_0_ + *g* = 0.1, and *R*_0_ = *ω*_0_/*μ*, *R*_∞_ = *κCω*_0_ with 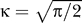 .

If *D* = 0, this is linear in *x* with slope *C*, and with *D* > 0, it is asymptotically linear as *x* → ∞. The exponential factor is important for small *x*, since it ensures that *b*_0_(*x*) is very flat, increasing only slowly near *x* = 0. In Appendix C of Text S1, it is shown that, for *D* > 0 and *C* sufficiently small, and with relative vitality given by Equation 12 (and for the more general forms considered in Appendix A of Text S1), *R*(*x*) in Equation 13 is monotonically decreasing in *x*, with *R*(*x*) → *κCω*_0_, a positive constant, as *x* → ∞ (where 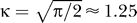 ). Clearly, if *R*(*x*) is decreasing and *Cω*_0_ ≥ 1, then *R*(*x*) > 1 for all *x*, and so there is a viable equilibrium population for every value of *x*, representing an evolutionary continuum from the most extreme compressed life history (*x* → ∞) to the most extreme spread, nonsenescent life history (*x* = 0). Examples of behaviors of *R*(*x*) for *b*_0_(*x*) in the family of Equation 14 are shown in Figure 2A–2C. Figure 2D shows an example in which *b*_0_(*x*) increases rapidly for small *x*, and then only slowly approaches its straight-line asymptote. The corresponding *R*(*x*) has a complicated shape, with two evolutionary optima, one near *x* = 0, and a more prominent one at a value near *x* = 1.

In terms of life-history constraints, the spreader-favoring birth functions impose developmental conditions that cause mutations away from the nonsenescent state *x* = 0 to have little impact on early adult recruitment compared to their impact on relative vitality.

This result shows that it is possible for an indefinite sequence of spreader mutations to invade, each one leading to an increase in population density, and eventually leading to the fitness-maximizing, nonsenescent state at *x* = 0. In Text S1, we show that this result holds under much more general assumptions on the form of relative vitality and initial recruitment function, but still with the fundamental life-history tradeoff expressed in terms of a senescence variable *x* governing the magnitude of the age-specific rate of senescence 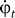 .

### Simulations

**Figure 3:**
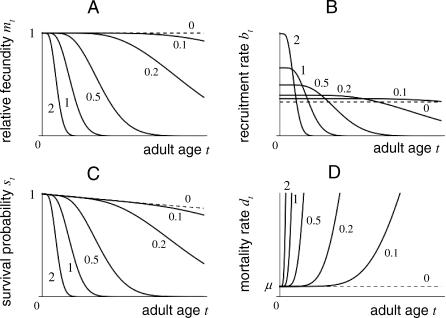
Graphs of Vital Rates (A) Relative fecundity functions *m_t_*(*x*) taking the form of Equation 15a at age *t* for *x* = 0, 0.1, 0.2, 0.5, 1, and 2, as indicated. (B) Total adult recruitment functions *b_t_*(*x*) = *b*_0_(*x*)*m_t_*(*x*), with *b*_0_(*x*) = 1 + *x*. (C) Survival functions *s_t_*(*x*) taking the form of Equation 15b. (D) Mortality rate functions 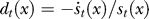 derived from Equation 15b. The senescence rate is of the age-dependent form 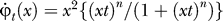 , with *n* = 5 (further detailed in Appendix A of Text S1). Other parameters are *α_b_* = *α_d_* = 0.5 and *μ* = 0.01. All graphs are on the same timescale.

For a given senescence rate 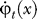 , we assume that relative vitality is partitioned between births and deaths as follows:

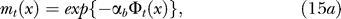


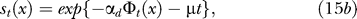
where 0 ≤ *α_b_*, *α_d_* ≤ 1 are fixed (age-independent) parameters with *α_b_* + *α_d_* = 1, *μ* = *μ*_0_ + *g* is the total age-independent mortality rate, and 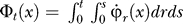 . A partition of this form is illustrated in Figure 3.

**Figure 4:**
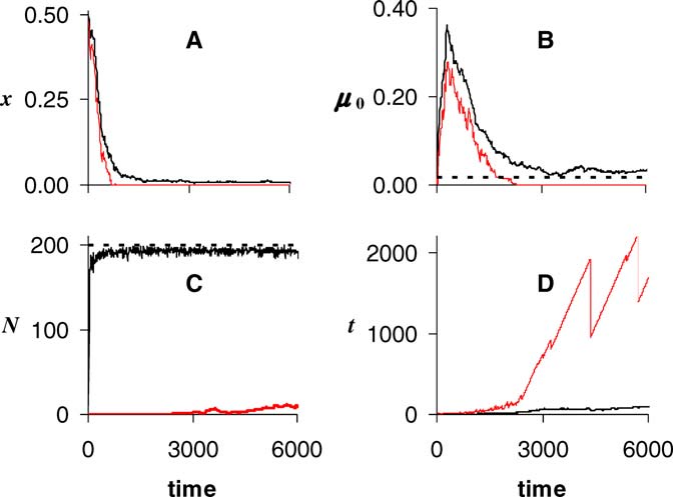
Simulated Adult Population in Presence of a Low Rate of Extrinsic Adult Mortality (A) Evolution over time in *x* (population mean and minimum), showing progressively later onset of vitality loss until most have negligible or zero senescence. (B) Concurrent reduction in *μ*_0_ (mean and minimum) until some have zero aging; the dashed line is the optimum *μ*_0_^*^ = 0.017, determined by the Marginal Value Theorem (see Appendix B of Text S1). (C) Adult population (upper black line) and intrinsic immortals (lower red); the dashed line is the carrying capacity *K*. (D) Adult age (mean and maximum) showing extended span for intrinsic immortals. Input parameter values: *α_b_* = *α_d_* = 0.5; random mutational increments of up to ±0.01 in *x* and *μ*_0_; δ*t* = 0.01; *B*_0_ = 10, *D*_0_ = 0.3; *ω*_0_ = *B*_0_ *μ*_0_/(*D*_0_ + *μ*_0_); *g* = 0.001; and *b*_0_(*x*) = *ω*_0_(1 + 0.2*xe* ^−1/*x*^).

Stochastic simulations were developed to exploit the Gaussian example of Equation 12 using a partition of the form of Equation 15a and 15b; i.e., with Φ*_t_*(*x*) = ½(*xt*)^2^ (see Methods below). Populations evolved towards smaller *x*, and thus slower senescence, given sufficiently small *C* and large *D* for Equation 14. Figure 4 shows an example of a population with extrinsic adult mortality set to a low value (small *g*), in addition to the extrinsic mortality imposed on juveniles by their inability to dislodge resident adults from any of the 200 habitable sites. The individuals making up the population were given life histories characterized by a relatively large *R*_0_ (as in Figure 2A), and an equal partition of senescence between birth rate and period survival (*α_b_* = *α_d_* = 0.5). The size of *x* and *μ*_0_ diminished rapidly (Figure 4A and 4B) after population size had equilibrated (Figure 4C), with eventual production of some intrinsically immortal individuals (Figure 4C, red line). These immortals did not senesce, because they had zero age-dependent loss of vitality (*x* = 0), nor did they age, because they had zero intrinsic mortality (*μ*_0_ = 0). They could never accumulate to displace all mortals, however, because each remained susceptible to extrinsic adult mortality. The mean value of *x* stabilized just above zero, probably due to an ever-increasing time to fixation in the population of ever-longer lived individuals. The simulation departed from the analytical model with respect to genetic variation, by allowing every offspring to carry a (small) mutation rather than having a linear sequence of mutation followed by fixation.

In further trials, setting *g* = 0 allowed immortals to accumulate in the population until they entirely filled it. Note that *g* = 0 does not imply no extrinsic mortality, only that the nonheritable components of mortality are concentrated in juvenile stages. Populations evolved negligible senescence even under high exogenous hazard, although a large *g* favored a high *ω*_0_ (as predicted in Appendix B of Text S1) with the consequence that intrinsic nonsenescent aging, *μ*_0_, was also high, and populations did not sustain intrinsic immortals. With new-adult recruitment functions of the Equation 14 type controlled by large *C* and small *D*, such as the *b*_0_(*x*) for Figure 2B and 2C, populations evolved towards larger *x*, and thus faster senescence.

Simulations were also undertaken with *g* and *K* varying stochastically (stochastic environment). Conclusions obtained for constant *g* and *K* were robust under these extensions, except that immortals evolved more rarely (see examples in Appendix B of Text S1).

## Discussion

The analyses and simulations have employed an ecologically realistic framework in which selection on the life-history strategy depends on the chances for recruitment into the adult population. This in turn depends on the life history of the other (resident) individuals in the population. Such a situation is a form of frequency-dependent selection: the fitness of a strategy cannot be assigned in absolute terms, but it depends on the strategies of the other individuals in the population. Frequency-dependent life-history evolution is an underexplored, yet fundamental, component of the ecology in which selection operates on organisms.

We have obtained a general set of frequency-dependent conditions under which spreader mutations invade in indefinite sequence from any senescent state (Results sections Model: Invasion of Mutations, and Example). These results extend previous optimization analyses [17,24,31,34] by showing that the process once initiated is not systematically vulnerable to counteracting compressor mutations, and is largely free of conditions on life history for a population regulated by density-dependent recruitment. Any organism that currently favors spreader mutations at recruitment-regulated equilibrium may continue to do so endlessly in an environment with constant exogenous hazard over evolutionary timescales (Figure 2A and 2B, but cf. Figure 2C and 2D). Although somatic growth that continues into early-adult ages strengthens selection on nonsenescence (Results section Model: Senescent and Nonsenescent Aging as Components of Vitality Loss), it is not a prerequisite for it (Appendices A and C of Text S1). This is an important finding because early-adult growth is itself a form of negative senescence, and so begs the question of what selection pressures may favor it. These predictions are confirmed by simulations in various environments, including stochasticity in exogenous hazard and in carrying capacity (Results section Simulations and Appendix B of Text S1).

Our analysis explicitly models developmental constraints through the functional form of *b*_0_(*x*), and environmental hazard with the constant extrinsic mortality rate *g*, allowing us to explore their influences on the direction of evolution. The runaway selection for reduced senescence arises directly from a model of organisms with strong inherent cost to reproduction expressed by a function *b*_0_(*x*) that increases relatively slowly with *x*, especially for small *x* (Results section Example). In effect, such organisms have developmental constraints that impact more strongly on reproductive rate than on reproductive longevity. In contrast, cases in which *b*_0_(*x*) increases relatively fast represent organisms for which an increment in early reproduction carries only a light inherent cost. Unsurprisingly, selection then favors incrementally earlier reproduction in the presence of exogenous hazard, as it does if the cost is delayed far into the future. We have illustrated this range of outcomes for various forms of *b*_0_(*x*) that can sustain viable populations across all *x*, and have one or more attractors at 0 ≤ *x* ≤ ∞, depending on the shape of the function (Figure 2). Although there always exists a class of functions *b*_0_(*x*) with the required properties to trigger runaway selection for reduced senescence (Appendix C of Text S1), these will certainly form a small subclass of the set of all possible *b*_0_ functions. It is difficult to characterize the relative size of this functional space mathematically, and understanding the biological significance of its characteristics is a theme for future work.

Senescent aging theory from Medawar [1] onward has emphasized the fundamental role of exogenous hazard in favoring early reproduction. Accordingly, our definition of adult mortality retains an explicit distinction of extrinsic from intrinsic sources, *g* from *μ*_0_, with a positive value of *μ*_0_ representing a decline in relative vitality due to nonsenescent aging (Results sections Model: Senescent and Nonsenescent Aging as Components of Vitality Loss, and Example). The predictions of our model for evolution of nonsenescence from senescence embrace both adult and juvenile susceptibility to extrinsic mortality. We obtain the novel result that evolution of nonsenescence from senescence is robust to the presence of exogenous adult mortality. A reduction in the realized adult lifespan tends instead to increase the age-independent component of vitality loss (nonsenescent aging). In the event that organisms adapt to hazards in gene-by-environment interactions, selection for early reproduction to hedge against future mortality risk will be offset by selection to reduce this risk. Our distinction of nonsenescent from senescent aging offers a reworking of George Williams' hypothesis [6] that populations subject to high exogenous hazard should have faster senescence, which has received mixed empirical support (reviewed in [35]). Williams et al. [35] point out that this hypothesis constitutes the principal tool for predicting senescence schedules, because no other environmental factor has been proposed to account for observed variation in senescence rates. However, Caswell [36] has recently demonstrated theoretically that exogenous mortality can have no effect on the age pattern of selection gradients. In consequence, there is now an absence of predictive tools to account for environmentally induced variation in senescence. Our model provides a new one, in terms of exogenous mortality favoring nonsenescent aging under density-dependent recruitment.

Most natural organisms pack their reproductive output into relatively short lives. Our model is consistent with this reality, which has a number of drivers. Individuals may frequently die from extrinsic causes, before the onset of senescence as we model it. This is evidenced in the natural rarity of age-related fecundity loss, and the rarity of degenerative disorders such as Alzheimer disease in nonhuman species. Moreover, compressor mutations are favored by conditions of density-independent fecundity, and a high *μ*_0_ is optimal under high extrinsic mortality. Even if such conditions exist only temporarily, the evolution of shorter lifespans will tend always to proceed more swiftly than the evolution of longer lifespans (with concomitantly slower turnover). Organisms with very long lifespans tend to be completely or virtually immobilized as adults, which guarantees the consistent density dependence in recruitment that underpins the model of runaway selection on spreader mutations. Many organisms are not so place-dependent, and therefore may be less consistently prone to density-dependent recruitment. Finally, negligible senescence may be too costly or mechanistically impossible for many organisms, due to developmental constraints [23].

The crowded, but stable, conditions predicted to favor runaway selection on spreader mutations may contribute to the extreme lifespan of the Ocean Quahog Clam (Arctica islandica). Individuals of this species can attain lifespans in excess of 200 y [16] in highly aggregated populations [37]. Our analysis predicts that selection will favor longer reproductive lifespan in response to severely limited recruitment opportunities, for example, if larvae can secure a foothold in suitable sediment only where space is released by an adult death.

Likewise for the Bristlecone Pine: populations that support the longest-lived trees occupy arid and exposed sites that afford few recruitment opportunities [38]. Trees continue producing cones with viable seeds throughout adult life [19], suggesting that seeds and seedlings may have short viability relative to adult lifespans of some 5,000 y. Under these conditions, we have shown how selection can favor adults that outlive their neighbors to set seed in their place. Although this direct benefit of reduced adult mortality may not apply to species with juveniles that can outlive the adult stage, for example in seed or seedling banks, even a low rate of juvenile mortality may suffice to meet the condition for spreader invasion if the attrition is spread over a long enough pre-adult phase.

Recruitment limitation is important in determining the population density of the exceptionally long-lived Harvester Ant Pogonomyrmex occidentalis, which has an average colony life expectancy of 17 y [39]. Because nests are static structures and new queens almost never colonize their own nest, there is a clear advantage to a resident queen outliving her neighbors to implant offspring in their place. Other highly place-dependent species capable of long lifespans include the sea anemones, for example, Anthopleura xanthogrammica with indeterminate somatic growth and an average longevity estimated to exceed 150 y [40].

Negligible senescence is recorded for a few organisms susceptible to high extrinsic mortality, such as the freshwater hydra with an intrinsic lifespan of at least 4 y [18]. Its sessile habit is likely to induce strong density impacts on juvenile recruitment. However, our model cannot directly account for the species' apparent lack of aging (even in one 30-y-old clone; D. Martínez, personal communication); i.e., zero *μ*_0_. Although hydra are susceptible to predation and stochastic hazards in their shallow freshwater habitat, we suggest that the population genotype may be supplied from a source population sheltered from hazard (i.e., zero *g*). This would be interesting to model theoretically and empirically by testing for source-sink population dynamics.

The idea that colonists inherit traits for a high rate of population increase *r* while slower maturity prevails at carrying capacity *K* [41,42] became an ecological paradigm in the 1960s and seemed to promise a scheme for life-history evolution [43]. Declarations that this scheme is flawed now span four decades [44–48], attesting to the popularity of *r*- and *K*-selection as a dichotomy that endures even in apparent defiance of reason. Two principal deficiencies are deemed to render the original idea of *K*-selection inoperable as a life-history theory: (1) it lacks a specific equation linking carrying capacity *K* to individual life-history traits; and (2) its life-history predictions ignore age structure. Empirical evidence for life-history correlates of *r*- and *K*-selection is consequently mixed [44,47,48]. Our model recognizes the role of the explicitly age-structured ELRS in defining *K* (Equation 3). Analysis of evolution in ELRS generates the novel prediction that recruitment limitation can suffice alone to drive an indefinite delay in senescent onset and even indeterminate generation length. This type of *K*-selection applies more to organisms with inherently more costly reproduction, which are also those most susceptible to senescence creeping into their genomes [4]. Nonsenescence can arise in hazardous environments, but intrinsic immortality requires the near absence of extrinsic adult mortality (though not of extrinsic juvenile mortality).

## Methods

Simulation of evolution in senescent and nonsenescent aging.

In the nonsenescent state, *x* = 0, the death rate (Equation 1) is constant, *d_t_* = *μ*_0_ + *g*, where *μ*_0_ is intrinsic mortality and *g* is extrinsic mortality. If *μ*_0_ = 0, there is, in addition, no nonsenescent aging (organisms are intrinsically immortal; section Model: Senescent and Nonsenescent Aging as Components of Vitality Loss), so we refer to *μ*_0_ as the aging parameter. The nonsenescent (*x* = 0) ELRS is *R*_0_ = *ω*_0_/(*μ*_0_ + *g*), and we assume that independent selective processes operate to maximize *R*_0_ through a (concave) tradeoff between *ω*_0_ and *μ*_0_. Maximization of *R*_0_ is then determined by a Marginal Value Theorem (see Appendix B of Text S1).

Asexual genotype life histories were defined by age-specific birth and death profiles derived from Equations 15(a and b) and 14 (derivations in Appendix B of Text S1). At each time step in the simulation, each individual was given a chance to produce offspring, then to die, and then for its offspring to recruit with small mutational increments or decrements to *x* and *μ*_0_. Two parameter constants, *K* and *g*, described the simulated environment, and a further five defined the organism inhabiting it: *K*, total carrying capacity of habitable sites for adults; *g*, extrinsic mortality acting equally on all adult ages; *B*_0_, *D*_0_, shape parameters for the positive concave tradeoff between the nonsenescent rates of new-adult recruitment *ω*_0_ and intrinsic mortality *μ*_0_ (as in Figure B.1 in Appendix B of Text S1); *α_b_*, partitioning of vitality between births and deaths (*α_d_* = 1 − *α_b_*, as in Equations 15); *C*, *D*, parameters for birth function *b*_0_(*x*) (as in Equation 14).

An array of size *K* = 200 was initially seeded with a population of ten just-matured recruits (age *t* = 0). These had nonzero values of the two evolvable parameters controlling vitality loss: *x* (defining the senescence rate) and *μ*_0_ (nonsenescent mortality rate). Each subsequent time step involved: adult reproduction; adult death; juvenile recruitment to empty sites; and juvenile inheritance of parental *x* and *μ*_0_ with small mutations. Life-history evolution was monitored for a range of input parameter values. In some simulations, the environmental parameters *K* and *g* were allowed to vary stochastically at each time step. Details are given in Appendix B of Text S1.

## Supporting Information

Text S1 Appendices (Appendix A) General theory—describes a class of age-dependent senescent rate functions that generalize the constant rate form considered in the main text, and which includes the effect of negative senescence. Examples of these more-general forms are developed.
(Appendix B) Simulated birth and death probabilities—describes the simulation model in more detail, the Marginal Value Theorem, and further simulation results, including for stochastic environments.
(Appendix C) Mathematical conditions for decreasing *R*(*x*)—contains technical mathematical proofs of conditions under which functions *b*_0_(*x*) can be found for which *R*(*x*) is monotonically decreasing in *x*.
(1.8 MB DOC)

## Abbreviations

ELRS: expected lifetime reproductive success
