# Supplementary material for: Density Dependence Triggers Runaway Selection of Reduced Senescence
Source: PLoS Comput Biol. 2007 Dec 28;3(12):e256. doi: 10.1371/journal.pcbi.0030256 (PMC2230684; doi:10.1371/journal.pcbi.0030256)
Supplement: Text S1 — (Appendix A) General theory—describes a class of age-dependent senescent rate functions that generalize the constant rate form considered in the main text, and which includes the effect of negative senescence. Examples of these more-general forms are developed. (Appendix B) Simulated birth and death probabilities—describes the simulation model in more detail, the Marginal Value Theorem, and further simulation results, including for stochastic environments. (Appendix C) Mathematical conditions for decreasing R(x)—contains technical mathematical proofs of conditions under which functions b 0(x) can be found for which R(x) is monotonically decreasing in x. (1.8 MB DOC) [file pcbi.0030256.sd001.doc]

# Density Dependence Triggers Runaway Selection of Reduced Senescence

### Robert M. Seymour and C. Patrick Doncaster

These Appendices contain further theoretical development and technical results to supplement the main text of this paper, and are arranged as follows.

*Appendix A.*– Describes a class of age-dependent senescent rate functions that generalise the constant rate form considered in the main text, and which includes the effect of negative senescence. Examples of these more general forms are developed.

*Appendix B.*– Describes the simulation model in more detail, the Marginal Value Theorem, and further simulation results including for stochastic environments.

*Appendix C.*– Contains technical mathematical proofs of conditions under which functions *b*_0_(*x*) can be found for which $R(x)$ is monotonically decreasing in *x*.

### Appendix A: General theory

##### Concepts

We represent the age-specific rate of senescence as the difference of two opposing forces, one promoting positive senescence (damage accumulation) and the other negative senescence (damage repair). Specifically, take to have the general form:

, (A1)

with $x\geq0$ a parameter, and:

- $\lambda_{+}(t)$: a non-negative, non-decreasing function of *t* with $\lambda_{+}(t)\to1$ as $t\to\infty$
- $\lambda_{-}(t)$: a non-negative, decreasing function of *t* with $\lambda_{-}(t)\to0$ as $t\to\infty$

The non-negative term $x^{2}\lambda_{+}(t)$ represents the rate of positive senescence, which is assumed to increase with age, and $\lambda_{-}(t)$ represents the rate of negative senescence, which is assumed to decrease with age. It follows from the above properties that:

. (A2)

That is, $x^{2}$is the *asymptotic rate of senescence* of very old adults. By forcing this variable to be non-negative, we preclude the possibility that the net effect of negative senescence persists to arbitrarily old ages. The best that can happen at large ages is a declining effect of negative senescence, which occurs when $x=0$, but for $x>0$ there is necessarily positive senescence at sufficiently old ages. The maximum rate of negative senescence, achieved by newly recruited adults, is $\lambda_{-}(0)$. Clearly there is no negative senescence at any adult age if$\lambda_{-}(0)=0$.

The general effect of introducing negative senescence, suitably partitioned between births and deaths, is to give an early-adult life-history phase of negative senescence, in which fecundity increases and mortality decreases with age, followed by a late adult phase of positive senescence, in which fecundity declines and mortality increases. This general pattern is illustrated in Fig A.1 for an example to be developed in detail later in this Appendix.

##### Components of age-specific senescence rate

More specifically: if $\lambda_{+}(0)=0$ and $\lambda_{-}(0)>0$, then there is an adult age $T(x)$ for which when $0\leq t<T(x)$, giving an early-adult life-history phase of negative senescence (somatic growth and rising fertility), and for which when $t>T(x)$, giving a later life-history phase of positive senescence.


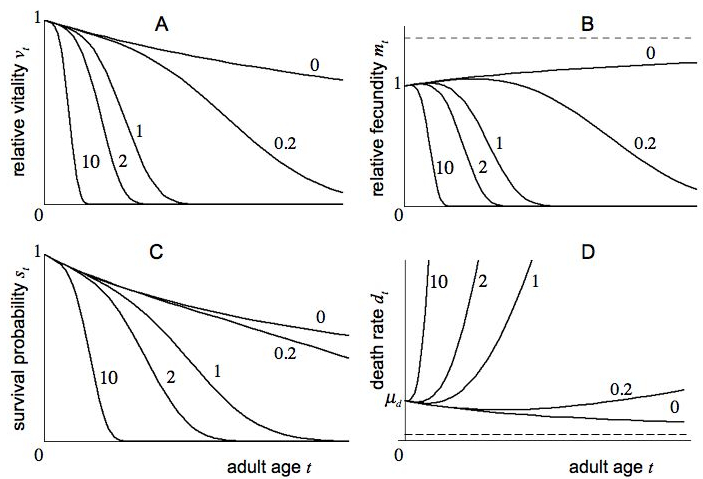


**Fig A.1.** Graphs of vital rates. (**A**) Relative vitality functions taking the form of Equation A11 at age *t* for *x* = 0, 0.2, 1, 10, as indicated. (**B**) Relative fecundity $m_{t}(x)$ given by a partition of the form of Equation A12a. The dashed line is the asymptote $m^{*}=(\eta_{d}/\eta)^{g/\eta}$ for the $x=0$ trajectory that has relative fecundity increasing throughout adult life. (**C**) Survival probabilities taking the form of Equation A12b. (**D**) Death rate functions derived from Equation A12b. The dashed line is the asymptote $\mu_{0}$ for the $x=0$ trajectory and $\mu_{d}=\mu_{0}+(\eta_{d}/\eta)g$. The parameters are: $n=2$, $k=10$, $\gamma_{0}=0$, $\mu_{0}=0.005$, $g=0.02$, $\eta=0.04$, $\eta_{d}=0.08$, $\alpha_{b}=0.9$, $\alpha_{d}=0.1$. All graphs are on the same timescale.

It follows from Equation A1 that the age-specific rate of loss of relative vitality is:

$\phi_{t}(x)=\phi_{0}+x^{2}\Lambda_{+}(t)-\Lambda_{-}(t)$, (A3)

where $\Lambda_{\pm}(t)=\int_{0}^{t} \lambda_{\pm}(s)ds$. The constant $\phi_{0}$ is the non-senescent component of the rate of vitality loss, which we assume is positive and independent of *x*. Finally, set

$\Phi_{t}(x)=x^{2}\int_{0}^{t} \Lambda_{+}(s)ds-\int_{0}^{t} \Lambda_{-}(s)ds$. (A4)

Thus, relative vitality is given by: $v_{t}(x)=\exp\left\{-\Phi_{t}(x)-\phi_{0}t\right\}$.

We allow the possibility that $\Lambda_{\pm}(t)$ also depend on *x*. This represents a tradeoff between the asymptotic rate of vitality loss and the timing of its approach to this level. Thus, we might expect a greater age-specific rate of vitality loss due to positive senescence as *x* increases. Similarly, the influence of negative senescence on the age-specific rate of vitality loss should decrease as the strength of the positive senescence effect increases with *x*. That is, we require:

(a) $\frac{\partial\Lambda_{+}(t)}{\partial x}\geq0$ (b) $\frac{\partial\Lambda_{-}(t)}{\partial x}\leq0$. (A5)

Clearly Equations A4 and A5 imply that $v'_{t}(x)<0$ for each $t>0$ and $x>0$, and hence that $S(x)=\int_{0}^{\infty} v_{t}(x)e^{-gt}dt$ is a monotonically decreasing function of *x*. In Appendix C we shall show that there exists a family of new-adult recruitment functions, $b_{0}(x)$, which are monotonically increasing in *x* with $b_{0}(0)=\omega_{0}>0$, for which $R(x)=b_{0}(x)S(x)$ is monotonically decreasing in *x*. In addition, these functions can be chosen so that $R(x)\to R_{\infty}$ as $x\to\infty$, with $R_{\infty}$ a positive constant. Thus, if $R_{\infty}\geq1$, then $R(x)>1$ for each $x\geq0$, and hence there is a possible viable equilibrium population for each *x* (main text, section 1), defining an evolutionary continuum as considered in the main text.

##### Example

We consider a model in which negative senescence acts to ameliorate the effect on vitality loss due to extrinsic mortality, for example through somatic growth allowing escape from predation and/or more effective food capture. For example, suppose that

$g-\Lambda_{-}(t)=g\frac{1}{1+\eta t}$. (A6)

This means that negative senescence has the effect of reducing the extrinsic mortality rate with increasing age, with the result that, if there were no other source of mortality, extrinsic mortality would eventually go to zero. Here, $\eta$ is a positive parameter measuring the rate at which extrinsic mortality decreases. Clearly there is no negative senescence effect if $\eta=0$.

We allow the possibility that $\eta=\eta(x)$ is a function of the senescence variable *x*. Such dependence would express a possible tradeoff between positive and negative senescence, as in Equation A5. Thus, as the controlling rate of positive senescence *x* increases, implying a rapid loss of vitality with age, the force of negative senescence, promoting vitality-increasing somatic and/or behavioural development, may decrease; i.e. $\eta(x)$is decreasing in *x*. However, nothing in what follows depends on such a tradeoff assumption, so we suppress this *x*-dependence from the notation.

It now follows from Equation A6 that the negative senescence rate is given by:

$\lambda_{-}(t)=\frac{g\eta}{(1+\eta t)^{2}}$. (A7)

For positive senescence, take $\lambda_{+}(t)$ to be a Hill function:

$\lambda_{+}(t)=\frac{(xt)^{n}}{k^{n}+(xt)^{n}}$, (A8)

for $n>0$, with $k>0$ a constant defining an age scaling factor which determines the age of onset of significant senescence. Thus, for *n* large, $\lambda_{+}(t)$ remains small for $t<\sim k/x$, but increases rapidly for $t>k/x$ (Fig A.2A). This represents delayed-onset senescence. Then we have:

$\Lambda_{+}(t)=\frac{k}{x}\theta_{n}(xt/k)$, $\int_{0}^{t} \Lambda_{+}(s)ds=\frac{k^{2}}{x^{2}}\Theta_{n}(xt/k)$, (A9)

where the functions $\theta_{n}(t)$ and $\Theta_{n}(t)$are defined explicitly in terms of standard special functions (see the final subsection of this Appendix, Equations A16 to A21 and Fig A.2B and C). It now follows from Equations A4, A6 and A9 that:

$\Phi_{t}(x)=k^{2}\Theta_{n}(xt/k)+\frac{g}{\eta}\ln\left(1+\eta t\right)-gt$, (A10)

and that relative vitality has the form:

$v_{t}(x)=\left(\frac{1}{1+\eta t}\right)^{\frac{g}{\eta}}\exp\left\{-k^{2}\Theta_{n}(xt/k)-(\phi_{0}-g)t\right\}$. (A11)

Notice that the limit as $\eta\to0$ of the first factor multiplying the exponential term is just $e^{-gt}$, which cancels with the $e^{gt}$ appearing in the exponential term. Thus, in this no-negative senescence limit, $v_{t}(x)$ is independent of the extrinsic mortality rate *g*.

We assume that relative vitality is partitioned between births and deaths as follows:

$m_{t}(x)=\left(\frac{1+\eta_{d}t}{1+\eta t}\right)^{\frac{g}{\eta}}\exp\left\{-\alpha_{b}k^{2}\Theta_{n}(xt/k)\right\}$, (A12a)

$s_{t}(x)=\left(\frac{1}{1+\eta_{d}t}\right)^{\frac{g}{\eta}}\exp\left\{-\alpha_{d}k^{2}\Theta_{n}(xt/k)-\mu_{0}t\right\}$, (A12b)

where:

$\alpha_{b}+\alpha_{d}=1$, $\phi_{0}=\mu_{0}$. (A13)

Here, $\mu_{0}$ is the intrinsic, non-senescent component of mortality. Note that in Equation A12b we have used the fact that $v_{t}(x)=m_{t}(x)s_{t}^{in}(x)$, and total survival is given by $s_{t}(x)=s_{t}^{in}(x)e^{-gt}$.

We assume that $\eta_{d}\geq\eta$, and that $\eta_{d}/\eta\to1$ sufficiently fast as $\eta\to0$; for example, $\eta_{d}=\eta\exp(\sigma\eta^{2})$ with $\sigma\geq0$. The limit as $t\to\infty$ of the factor multiplying the exponential in Equation A12a is $m^{*}=\left(\eta_{d}/\eta\right)^{g/\eta}$, which is greater than 1 if $\eta_{d}>\eta>0$. In this case, this factor is monotonically increasing in *t* from 1 at *t* = 0 to the asymptotic value $m^{*}$. This increase represents the negative senescent effect on reproduction – an increase in reproductive capacity acquired with increasing age. On the other hand, the exponential term in Equation A12a represents the depressive effect on reproduction of a deterioration in physiological condition with age. Note that, with $\eta_{d}$ given as above, we have $m^{*}=\exp\{g\eta\sigma\}\to1$ as $\eta\to0$. Thus, there is no negative-senescence enhancement of reproduction in this limit, as expected.

The term multiplying the exponential in Equation A12b represents the effect on survival of extrinsic mortality. Note that, as $\eta\to0$, this term tends to $e^{-gt}$, which recovers the extrinsic mortality effect on survival.

The partition defined by Equations A12a, b is illustrated in Fig A.1.

##### Relevant special functions

We require the class of special functions known as *hypergeometric functions*, defined by:

$_{2}F_{1}(a,b;c;x)=\sum_{k=0}^{\infty} \frac{(a)_{k}(b)_{k}}{(c)_{k}}\cdot\frac{x^{k}}{k!}$, (A14)

where $(a)_{0}=1$ and $\left(a\right)_{k}=a\left(a+1\right)\ldots\left(a+k-1\right)$ for $k\geq1$ (Whittaker & Watson, 1978, Chapter XIV).

The functions $\theta_{n}(t)$ and $\Theta_{n}(t)$:

For $n>0$, define

$\theta_{n}(t)=\int_{0}^{t} \frac{s^{n}}{1+s^{n}}ds$. (A15)

This can be represented explicitly in terms of hypergeometric functions:

$\theta_{n}(t)=\frac{t^{n+1}}{n+1}\cdot{}_{2}F_{1}\left(1,1+\frac{1}{n};2+\frac{1}{n};-t^{n}\right)$. (A16)

[*Mathematica* 5.0 software package.] Now define:

$\Theta_{n}(t)=\int_{0}^{t} \theta_{n}(s)ds$. (A17)

Again, this can be represented in terms of hypergeometric functions:

$\Theta_{n}(t)=t^{n+2}\left\{\frac{1}{n+1}\cdot{}_{2}F_{1}\left(1,1+\frac{1}{n};2+\frac{1}{n};-t^{n}\right)-\frac{1}{n+2}\cdot{}_{2}F_{1}\left(1,1+\frac{2}{n};2+\frac{2}{n};-t^{n}\right)\right\}$.

(A18)

[*Mathematica* 5.0 software package.] Finally, for $k>0$, define $\theta_{n}(t;k)$ and $\Theta_{n}(t;k)$ by:

$\theta_{n}(t;k)=\int_{0}^{t} \frac{s^{n}}{k^{n}+s^{n}}ds$, $\Theta_{n}(t;k)=\int_{0}^{t} \theta_{n}(s;k)ds$. (A19)

Then we have:

$\theta_{n}(t;k)=k\theta_{n}(t/k)$, $\Theta_{n}(t;k)=k^{2}\Theta_{n}(t/k)$. (A20)

Thus, a non-unity value of *k* represents a rescaling of time *t*.

It is worth noting that, in the limit $n\to\infty$, we have $\lambda_{+}(t)=H[t-1]$, a Heavyside function, and hence

$\theta_{\infty}(t)=(t-1)H[t-1]$, . (A21)

Graphs of $t^{n}/(1+t^{n})$, $\theta_{n}(t)$, $\Theta_{n}(t)$ and the relative vitality component $\exp\left\{-k^{2}\Theta_{n}(xt/k)\right\}$ for various *x* are illustrated in Fig A.2 below.


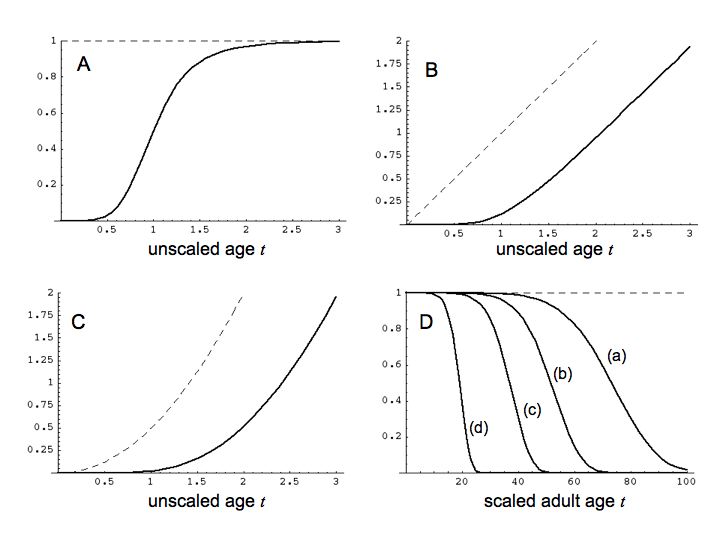


**Fig A.2**. (**A**) Graph of $t^{n}/(1+t^{n})$. The dashed horizontal line is the asymptote. (**B**) Graph of $\theta_{n}(t)$. The dashed curve is the function obtained by integrating under the asymptote in A; i.e. *t*. (**C**) Graph of $\Theta_{n}(t)$. The dashed curve is the function obtained by integrating under the dashed curve in B; i.e. . (**D**) Graphs of the relative vitality component $\exp\left\{-k^{2}\Theta_{n}(xt/k)\right\}$ for various *x*: (a) *x* = 0.2, (b) *x* = 0.3, (c) *x* = 0.5, (d) *x* = 1. The dashed horizontal line is obtained by taking *x* = 0. In all graphs, *n* = 5, and in D the age-scaling factor is *k* = 25.

### References

Whittacker, E.T. and Watson, G.N. (1978) *A Course of Modern Analysis*. Cambridge University Press, 608 pp.

### Appendix B: Simulated birth and death probabilities

For the simulations described in section 5 of the main text (Fig. 4) and further in this appendix, an array of *K* = 200 possible settlement sites was initially seeded with a population of 10 just-matured recruits (age *t* = 0), with no more than one individual at a site. These individuals had non-zero values of the two evolvable parameters controlling vitality loss: *x* (age-dependent senescence) and *μ*_0_ (age-independent ageing). Each subsequent time-step from *t* to $t+\delta t$ involved the following operations on each adult in the population:

1. *Reproduction*. – Defined by the birth rate, as explained below.

2. *Adult death*. – Derived from survival function , as explained below.

3. *Juvenile recruitment*. – For each parent in turn, one offspring was sent to a random position in the array of habitable sites, at which it would immediately die if already occupied, or recruit if empty. The process was repeated for each of the parent’s offspring.

4. *Inheritance*. – The recruiting juvenile inherited its parent’s *x* and *μ*_0_ with mutational increments or decrements to both, each of random magnitude ≤ 0.01.

5. *Randomization of locations*. – The order of surviving residents and new recruits was randomized in the array, in preparation for stepping through it in the next time-step.

Here, $\mu=\mu_{0}+g$ with *g* extrinsic mortality acting uniformly on all adult ages. The environment in which organisms evolve is therefore characterized by the two parameters *K* and *g*. The remaining parameters characterize features of the organism. The time step was taken to be *δt* = 0.1. Further details of these simulation steps are given below.

##### Offspring Production from t to t + δt

For given *x*, birth *rate* at age *t* is:

. (B1)

This means that $b_{t}(x)\delta t$ is the probability that an organism produces an offspring in the *infinitesimal* time interval *t* to $t+\delta t$ (under the assumption that $\delta t$ is so small that the probability of producing two or more off spring is of order $\delta t^{2}$, which is negligible). This is generalized Poisson (with time-dependent frequency). If

$B_{t}^{s}(x)=\int_{t}^{t+s} b_{\tau}(x)d\tau$,

then it can be shown that the probability that the organism produces $k\geq0$ offspring in the finite time interval *t* to $t+s$ is:

$P_{t,k}^{s}(x)=\frac{\left(B_{t}^{s}(x)\right)^{k}}{k!}\exp\left\{-B_{t}^{s}(x)\right\}$. (B2)

In the context of the simulations, take $s=\delta t=0.1$. This is small, but not infinitesimal. However, rather than evaluating the integrals explicitly, we approximate $b_{t}(x)$ by a piecewise constant function. That is, we assume that $b_{t}(x)$ is constant over the interval *t* to $t+\delta t$(with *t* an integer multiple of $\delta t$). Given that $\delta t$ is not too large, this is not unreasonable. This approximation means that we can write

$B_{t}^{\delta t}(x)=\int_{t}^{t+\delta t} b_{\tau}(x)d\tau\approx b_{t}(x)\delta t$,

and hence from (B2)

$P_{t,k}(x)=P_{t,k}^{\delta t}(x)=\frac{\left(b_{t}(x)\delta t\right)^{k}}{k!}\exp\left\{-b_{t}(x)\delta t\right\}$.

Substituting from (B1) for $b_{t}(x)$, then gives:

. (B3)

This is the Poisson distribution of *per capita* offspring production between *t* and δ*t* (i.e. one time step of the simulation). This is the formula used in the simulations of section 5 of the main text.

##### Probability of Death from t to t + δt

The probability of survival from time *t* to time $t+\delta t$ (given that the organism has survived to time *t*) is:

$p_{t}(x)=\frac{s_{t+\delta t}(x)}{s_{t}(x)}$.

With , this gives:

,

which can be expanded to:

.

If $\delta t$ is small ($\delta t=0.1$ in the simulations), terms of order $(\delta t)^{2}$ and above can be neglected to obtain the approximation:

$p_{t}(x)=\exp\left\{-\left[\alpha_{d}x^{2}t+\mu\right]\delta t\right\}$. (B4)

This is the form used in the simulations of section 5 of the main text. The probability that an organism dies in the interval *t to* $t+\delta t$ is then $1-p_{t}(x)$.

##### The Marginal Value Theorem

Consider the situation of constant birth and intrinsic death rates $\omega_{0}$ and $\mu_{0}$. Spreader or compressor mutations could affect the value of these also, independently of the senescence variable *x*. We assume that mutations move $\omega_{0}$ and $\mu_{0}$ along some trade-off (cost of reproduction) curve $\omega_{0}=B(\mu_{0})$, with $B(0)=0$ and $B(\mu_{0})$ concave increasing (see Fig. B.1). Then we can obtain optimal values $\mu_{0}^{*}$ and $\omega_{0}^{*}=B(\mu_{0}^{*})$ which maximize $R_{0}=\omega_{0}/(\mu_{0}+g)$ from the *Marginal Value Theorem*. This states that these optimal values are determined by the unique solution of:

$B'(\mu_{0}^{*})=\frac{\omega_{0}^{*}}{\mu_{0}^{*}+g}$. (B5)

The proof is trivial and well known (e.g., Bulmer 1994). The theorem is illustrated graphically in Figure B.1.

For example, if $B(\mu)=B_{0}\mu/(D_{0}+\mu)$ for some positive constants $B_{0}$ and $D_{0}$, then:

$\mu_{0}^{*}=\sqrt{D_{0}g}$, $\omega_{0}^{*}=\frac{B_{0}\sqrt{g}}{\sqrt{D_{0}}+\sqrt{g}}$, $R_{0}^{*}=\frac{B_{0}}{\left(\sqrt{D_{0}}+\sqrt{g}\right)^{2}}$. (B6)

This is the form of trade off curve used in the simulations of section 5 of the main text.


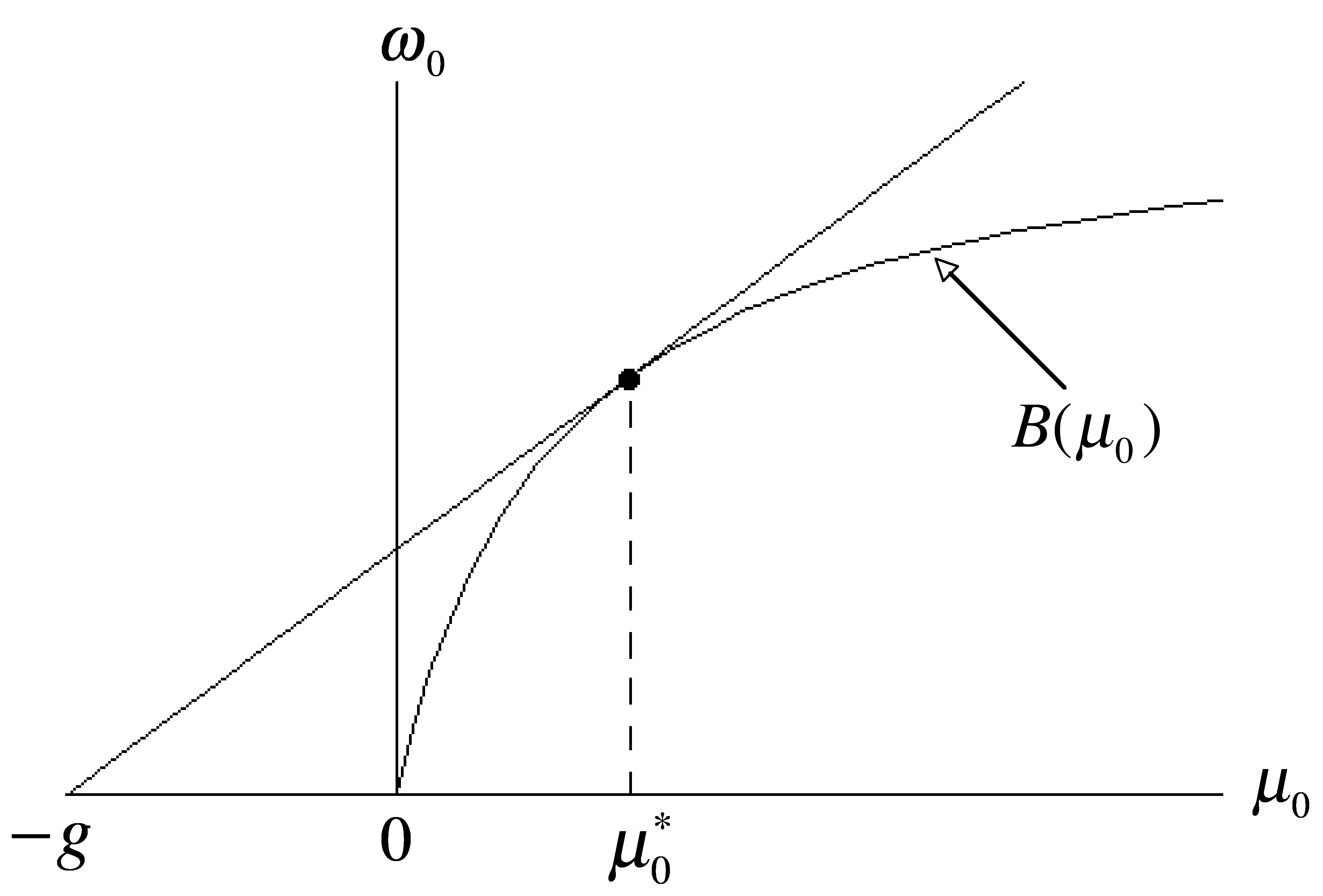


**Fig B.1.** The marginal value theorem determining the optimizing values $\mu_{0}^{*}$ and $\omega_{0}^{*}=B(\mu_{0}^{*})$.

##### Environmental Stochasticity

The main model had a constant environment defined by the value of *g* (extrinsic mortality) and the size of *K* (carrying capacity of adults). The simulation was extended as outlined below to investigate impacts of environmental stochasticity by varying *g* on a lognormal distribution, and *K* on a normal distribution, every *n*th time step. Any drop in carrying capacity incurred a proportionate impact on the resident adults. Thus a drop in *K* from 200 to 150 entailed a 25% drop in the number of adults (chosen at random for removal from the population).

The environmental stochasticity had no perceptible effect on the runaway selection for smaller *x*. Varying *g* alone gave outputs indistinguishable from the constant environment case shown in Figure 4 of the main text, with respect to the evolution of *x* and *μ*_0_, and the appearance of immortals, even for variances that encompassed frequent 5-fold increases given by ln(variance) = 1. This negligible impact of stochasticity in *g* applied whether *g* varied every 10 or every 100 time steps. However, a larger ln(variance) = 5 in *g* did prevent the appearance of immortals by sufficiently raising the mean *μ*_0_ to virtually eliminate the occurrence of any individuals with *μ*_0_ = 0. The addition of stochastic variation to *K* as well as to *g* also reduced the appearance of immortals by raising the mean *μ*_0_.

Figure B.2 shows outputs from the model with parameter values as in Figure 4 in the main text, except for the infrequent occurrence of relatively low-level fluctuations in both *g* and *K*. The environmental stochasticity has no effect on the evolution of *x* (graph A), but the fluctuations in *K* raise *μ*_0_ above its optimum set by the marginal value theorem (graph B), causing a reduced frequency of intrinsic immortals (graph C) and consequent reduction in maximum lifespan in the population (graph D). The occurrence of more frequent and larger environmental stochasticity further reduces the frequency of immortals without influencing evolution in *x*. Figure B.3 shows the population supporting the appearance of single immortals only twice and briefly during the simulation.

**Fig. B.2.** Simulated evolution of negligible senescence in the presence of relatively slow and low environmental stochasticity. Fluctuations occur at every 500 time steps in *g* and *K*, with *g* having ln(variance) = 0.5 on a normal distribution around a mean of ln(0.001) and *K* having variance = 50 on a normal distribution around a mean of 200; other parameter values as main text Figure 4. Red lines in A-C show minima below black-line means, and maximum in D above black-line mean. Blue line in C shows carrying capacity above black-line population size, *N*.

**Fig. B.3.** Simulated evolution of negligible senescence in the presence of relatively fast and high environmental stochasticity. Fluctuations occur at every 100 time steps in *g* and *K*, with *g* having ln(variance) = 1 on a normal distribution around a mean of ln(0.001) and *K* having variance = 400 on a normal distribution around a mean of 200; other parameter values and lines as Figure B.2.

### Reference

Bulmer, M. 1994. Theoretical evolutionary ecology. Sinauer, Sunderland Massachusetts.

### Appendix C: Mathematical conditions for decreasing *R*(*x*)

In this appendix we give mathematical proofs of three results. First, that the class of new-adult recruitment functions defined by Equation 14 in the main text contains a subclass which yields decreasing $R(x)$ for the Gaussian relative vitality function of Equation 12. Second that, for more general representations of relative vitality, such as those considered in Appendix A, Equation A1, there is always a class of new-adult recruitment functions, with appropriate properties, for which $R(x)$ is decreasing. Third, we show that the presence of negative senescence can only promote a decreasing $R(x)$ relative to its absence.

##### A class of new-adult recruitment functions

Consider the explicit class of new-adult recruitment functions discussed in the main text:

$b_{0}(x)=\omega_{0}\left(1+Cxe^{-\frac{D}{x}}\right)$. (C1)

We shall show that this family contains examples for which $R(x)$is decreasing and $R(x)\to R_{\infty}$, a positive constant, as $x\to\infty$. We prove the following result claimed in the main text.

PROPOSITION C.1. For each $D>0$, there is a range of positive values, $0<C<C^{*}$, such that $R(x)=b_{0}(x)S(x)$, determined by the Gaussian relative vitality function of main text Equation 12, is monotonically decreasing in *x*, with as $x\to\infty$.

*Proof*. First note that

. (C2)

[*Mathematica* 5.0 software package.] Clearly $S(x)$is positive and monotonically decreasing, with $S(0)=1/\mu$ and $S(x)\to0$as $x\to\infty$. Furthermore

$\frac{R'(x)}{R(x)}=\frac{b'_{0}(x)}{b_{0}(x)}+\frac{S'(x)}{S(x)}$. (C3)

Thus, $R'(x)<0$ for all positive *x* if and only if the right hand function in (C3) is negative.

Let $z=\mu/2x$, and define

$F(z)=\sqrt{\pi}z\exp(z^{2})\left\{1-\text{erf}(z)\right\}$, (C4a)

so that $S(x)=(1/\mu)F(z)$. Then $F(z)$ is non-negative, monotonically increasing, with $F(z)\to1$ as $z\to\infty$. We also easily establish the following properties of the related functions:

$\sqrt{\pi}z\left(\frac{1}{F(z)}-1\right)$ is positive, monotonically decreasing from value 1 at $z=0$ to asymptote 0 as $z\to\infty$. (C4b)

$z^{2}\left\{1-2z^{2}\left(\frac{1}{F(z)}-1\right)\right\}$ is non-negative, monotonically increasing from value 0 at $z=0$ to asymptote 1 as $z\to\infty$. (C4c)

[Derived using the *Mathematica* 5.0 software package.]

Define

$G(z)=1+\sigma\frac{1}{z}\exp\left(-\rho z\right)$, ($\sigma,\rho>0$). (C5)

Then $R(x)=R_{0}G(z)F(z)$ for and $\rho=(2/\mu)D$, with $R_{0}=\omega_{0}/\mu$. Since $z'(x)=-\mu/2x^{2}$, it follows from (C3) that $R'(x)<0$if and only if

$\frac{G'(z)}{G(z)}+\frac{F'(z)}{F(z)}>0$ (C6)

for all positive $z$.

We have $G'(z)=-\left(1+\frac{1}{z}\right)\left\{G(z)-1\right\}$ and:

$\frac{F'(z)}{F(z)}=\frac{1}{z}\left\{1-2z^{2}\left(\frac{1}{F(z)}-1\right)\right\}$, (C7)

Consider:

$$G(z)\left\{\frac{G'(z)}{G(z)}+\frac{F'(z)}{F(z)}\right\}$$

$$=\frac{F'(z)}{F(z)}G(z)+G'(z)$$

$=\frac{1}{z}G(z)-2z\left(\frac{1}{F(z)}-1\right)G(z)+\left(1+\frac{1}{z}\right)-G(z)-\frac{1}{z}G(z)$ from (C7)

$$=-2z\left(\frac{1}{F(z)}-1\right)G(z)+\left(1+\frac{1}{z}\right)-G(z)$$

$=\left(1+\frac{1}{z}\right)-\left\{1+2z\left(\frac{1}{F(z)}-1\right)\right\}\left\{1+\sigma\frac{1}{z}\exp(-\rho z)\right\}$ from (C5)

$$=\left(1+\frac{1}{z}\right)-\left\{1+2z\left(\frac{1}{F(z)}-1\right)\right\}-\sigma\frac{1}{z}\left\{1+2z\left(\frac{1}{F(z)}-1\right)\right\}\exp(-\rho z)$$

$$=\frac{1}{z}\left\{1-2z^{2}\left(\frac{1}{F(z)}-1\right)-\sigma\left[1+\frac{2}{\sqrt{\pi}}\cdot\sqrt{\pi}z\left(\frac{1}{F(z)}-1\right)\right]\exp(-\rho z)\right\}$$

$$\geq\frac{1}{z}\left\{1-2z^{2}\left(\frac{1}{F(z)}-1\right)-\sigma\left(1+\frac{2}{\sqrt{\pi}}\right)\exp(-\rho z)\right\}$$

because $\sqrt{\pi}z\left(1/F(z)-1\right)$ is a monotonically decreasing function with maximum value 1 at $z=0$ by (C4b). It therefore follows from this estimate that there is a range of positive values, $0\leq\sigma<\sigma^{*}(\rho)$, for which (C6) holds for all positive $z$, provided the function

$H(z)=\left\{1-2z^{2}\left(\frac{1}{F(z)}-1\right)\right\}\exp(\rho z)$.

is bounded away from 0 for positive $z$. This is always the case when $\rho>0$. To see this, note that $H(0)=1$ from (C4b), and we can write:

$H(z)=z^{2}\left\{1-2z^{2}\left(\frac{1}{F(z)}-1\right)\right\}\times\frac{1}{z^{2}}\exp(\rho z)$.

The first factor is positive and $\to1$ as $z\to\infty$ by (C4c), and the second factor is positive and $\to\infty$ as $z\to\infty$. Hence, $H(z)$ is positive for all $z\geq0$ and $H(z)\to\infty$ as $z\to\infty$ for any $\rho>0$. It follows from this that $H(z)$ is bounded away from 0. This shows that $R(x)$ is monotonically decreasing in *x* for sufficiently small *C*.

The limit $x\to\infty$

We have $R(x)=R_{0}G(z)F(z)$, and hence $R_{\infty}=R_{0}\lim_{z\to0}\left[G(z)F(z)\right]$. From Equations C4a and C5, we have $F(0)=0$, $G(0)=1$ and hence:

$$\lim_{z\to0} \left[G(z)F(z)\right]=\sqrt{\pi}\sigma\lim_{z\to0} \left[\frac{1}{z}\exp(-\rho z)\times z\exp(z^{2})\left\{1-\text{erf}(z)\right\}\right]$$

$$=\sqrt{\pi}\sigma\lim_{z\to0} \left[\exp(z^{2}-\rho z)\left\{1-\text{erf}(z)\right\}\right]$$

$$=\sqrt{\pi}\sigma$$

Thus, when , as required. This completes the proof of the proposition. 🞏

##### General new-adult recruitment functions

Define:

$S(x)=\int_{0}^{\infty} v_{t}(x)e^{-gt}dt$, (C8)

so that $R(x)=b_{0}(x)S(x)$ (see main text, Equation 13). It follows from Appendix A, Equations A4 and A5, that $S'(x)<0$ for $x>0$. We require to find conditions on the (increasing) function $b_{0}(x)$ so that $R'(x)<0$ for $x>0$.

First note that $-S'(x)/S(x)$ is an increasing function of *x*. Consider the class of functions:

$b_{0}(x)=\omega_{0}\exp\left\{-\int_{0}^{x} \psi(z)\left(\frac{S'(z)}{S(z)}\right)dz\right\}$, (C9)

where $\psi(x)$ is any non-decreasing function satisfying $0\leq\psi(x)<1$. Then $b_{0}(0)=\omega_{0}$, $b_{0}(x)$ is an increasing function of *x*, and:

$$\frac{R'(x)}{R(x)}=\frac{b'_{0}(x)}{b_{0}(x)}+\frac{S'(x)}{S(x)}=-\left(1-\psi(x)\right)\left(\frac{-S'(x)}{S(x)}\right)<0$$

for $x>0$, since $\psi(x)<1$. Thus, the family of new-adult recruitment functions (C9) satisfies the required conditions. This gives very general conditions under which $R(x)$ is decreasing.

The limit $x\to\infty$

We shall show that $\psi(x)$ can be chosen so that, not only is $R(x)$ monotonically decreasing, but also $R(x)\to R_{\infty}$ as $x\to\infty$ for some constant $R_{\infty}>0$. Hence, if $R_{\infty}$ is sufficiently large, $R(x)>1$ for all $x\geq0$. This implies that a viable population is possible for every *x*.

Under our assumptions we have $\psi(x)\to\psi_{\infty}\leq1$ and $S(x)\to S_{\infty}\geq0$ as $x\to\infty$. Take any $\psi(x)$ satisfying the additional condition:

$\psi(x)\geq1-\frac{S(x)}{S(X)}$ for all $x\geq X$, (C10)

with $X\geq0$ a fixed constant. Then, taking limits as $x\to\infty$, we have $1-S_{\infty}/S(X)\leq\psi_{\infty}\leq1$. Note that $\psi_{\infty}=1$ if $S_{\infty}=0$. It is straightforward to deduce from Equations C9 and C10 that, for $x\geq X$:

$R(x)\geq R(X)\exp\left\{-\left(1-\frac{S_{\infty}}{S(X)}\right)\right\}>0$.

[See below for a derivation.] Since $R(x)$ is positive and monotonically decreasing for $x\geq0$, it follows that $R(x)\to R_{\infty}$ as $x\to\infty$, for some positive constant $R_{\infty}$ with:

$R_{\infty}\geq R(X)\exp\left\{-\left(1-\frac{S_{\infty}}{S(X)}\right)\right\}$. (C11)

Since $S_{\infty}\geq0$, we have $R_{\infty}\geq R(X)e^{-1}$. If $R_{\infty}\geq1$, then $R(x)>1$ for all $x\geq0$, and hence there is a viable equilibrium population of organisms for any $x\geq0$.

Derivation of the estimate C11

$$b_{0}(x)=\omega_{0}\exp\left\{\int_{0}^{x} \psi(z)\left(\frac{-S'(z)}{S(z)}\right)dz\right\}$$

$\geq\omega_{0}\exp\left\{\int_{0}^{X} \psi(z)\left(\frac{-S'(z)}{S(z)}\right)dz+\int_{X}^{x} \left(1-\frac{S(z)}{S(X)}\right)\left(\frac{-S'(z)}{S(z)}\right)dz\right\}$ from (C10)

$$=b_{0}(X)\exp\left\{-\int_{X}^{x} \left(\frac{S'(z)}{S(z)}\right)dz+\frac{1}{S(X)}\int_{X}^{x} S'(z)dz\right\}$$

$$=b_{0}(X)\exp\left\{\ln\left(\frac{S(X)}{S(x)}\right)-\left(1-\frac{S(x)}{S(X)}\right)\right\}$$

$$=b_{0}(X)\left(\frac{S(X)}{S(x)}\right)\exp\left\{-\left(1-\frac{S(x)}{S(X)}\right)\right\}$$

$\geq b_{0}(X)\left(\frac{S(X)}{S(x)}\right)\exp\left\{-\left(1-\frac{S_{\infty}}{S(X)}\right)\right\}$.

Thus

$R(x)=b_{0}(x)S(x)\geq R(X)\exp\left\{-\left(1-\frac{S_{\infty}}{S(X)}\right)\right\}$,

from which the estimate C11 follows. 🞏

##### The role of negative senescence

Here we show that the presence of negative senescence in the form of a non-zero $\lambda_{-}(t)$in Equation A1 is more conducive to a decreasing $R(x)$ than its absence. To do this we denote by $R_{0}(x)$ the ELRS obtained from Equation A1 by setting $\lambda_{-}(t)=0$; i.e. by removing the influence of negative senescence. We shall prove the following result.

PROPOSITION C.2*.* If $b_{0}(x)$ is an initial birth function for which $R_{0}(x)$ is monotonically decreasing, then $b_{0}(x)$ is an initial birth function for which $R(x)$ is monotonically decreasing. Furthermore,$R(x)\geq R_{0}(x)$.

*Proof*. From Equation A4, we have:

$\Phi_{t}(x)=\phi_{0}t+x^{2}\int_{0}^{t} \Lambda_{+}(s)ds-\int_{0}^{t} \Lambda_{-}(s)ds\leq\phi_{0}t+x^{2}\int_{0}^{t} \Lambda_{+}(s)ds=\Phi_{0,t}(x)$,

and from Equation A5:

$\Phi'_{t}(x)=2x\int_{0}^{t} \Lambda_{+}(s)ds-\int_{0}^{t} \frac{\partial}{\partial x}\Lambda_{-}(s)ds\geq2x\int_{0}^{t} \Lambda_{+}(s)ds=\Phi'_{0,t}(x)$.

Thus:

$S(x)=\int_{0}^{\infty} \exp\left\{-\Phi_{t}(x)\right\}dt\geq\int_{0}^{\infty} \exp\left\{-\Phi_{0,t}(x)\right\}dt=S_{0}(x)$,

and

$-S'(x)=\int_{0}^{\infty} \Phi'_{t}(x)\exp\left\{-\Phi_{t}(x)\right\}dt\geq\int_{0}^{\infty} \Phi'_{0,t}(x)\exp\left\{-\Phi_{0,t}(x)\right\}dt=-S'_{0}(x)$.

It follows that:

$\frac{S'(x)}{S(x)}\leq\frac{S'_{0}(x)}{S_{0}(x)}$,

and hence:

$\frac{R'(x)}{R(x)}=\frac{b'_{0}(x)}{b_{0}(x)}+\frac{S'(x)}{S(x)}\leq\frac{b'_{0}(x)}{b_{0}(x)}+\frac{S'_{0}(x)}{S_{0}(x)}=\frac{R'_{0}(x)}{R_{0}(x)}$.

Thus, if $b_{0}(x)$ is a new-adult recruitment function for which $R'_{0}(x)<0$, then it is also a new-adult recruitment function for which $R'(x)<0$. Clearly, $R(x)=b_{0}(x)S(x)\geq b_{0}(x)S_{0}(x)=R_{0}(x)$. This proves the proposition. 🞏
